# Correlation between functional disability and quality of life among rural elderly in Anhui province, China: a cross-sectional study

**DOI:** 10.1186/s12889-021-12363-7

**Published:** 2022-02-25

**Authors:** Min Zhang, Weizheng Zhu, Xinran He, Yuyang Liu, Qian Sun, Hong Ding

**Affiliations:** Department of Health Service Management, School of Health Management, Anhui Medical University, No. 81 Meishan Road, Hefei, 230032 Anhui China

**Keywords:** Quality of Life, Rural elderly, Functional disability, China

## Abstract

**Objective:**

We aimed to explore the correlation between functional disability and quality of life (QoL). And exploring the interaction of functional disability, basic demographic characteristics and health-related information on QoL among the rural elderly in Anhui Province.

**Methods:**

This study used multi-stage stratified cluster sampling in rural Anhui Province from January to July 2018 to conduct a cross-sectional survey of older adults who met the inclusion criteria. The Five-Dimensional European Quality Of Health Scale (EQ-5D) and the WHO Disability Assessment Schedule (WHODAS2.0) scale were used to evaluate the QoL and functional disability, and the basic demographic characteristics of the survey subjects were also collected. Using binary logistic and Classification and regression tree model (CART) models to analyze the data, explore the relationship between functional disability and QoL in the elderly.

**Results:**

A total of 3491 older adults were included in the survey, and 3336 completed the entire survey, with an effective response rate of 95.56%. After adjusting for covariables, those who had limited in dimension of mobility (AOR=2.243, 95%CI: 1.743-2.885), getting along (AOR=1.615, 95%CI: 1.173-2.226), life activities (AOR=2.494, 95%CI:1.928-3.226), and social participation (AOR=2.218, 95%CI: 1.656-2.971) had a lower QoL. However, the dimension of cognition (AOR=0.477, 95%CI: 0.372-0.613) is a protective factor for QoL. Additionally, we also observe that 96.3% of those who were unemployed and limited in both mobility and life activities dimensions had a lower QoL, but among those who were robust in both mobility and social participation dimensions and not suffer from chronic diseases, 56.3% had a higher QoL.

**Conclusions:**

Our findings indicate that special attention should be paid to the elderly who are unemployed, have limited in cognition, getting along, social participation, mobility, life activities and cognition robust to improve their QoL. This research is of great significance for formulating targeted strategies and measures to improve the QoL for rural elderly.

**Supplementary Information:**

The online version contains supplementary material available at 10.1186/s12889-021-12363-7.

## Introduction

Aging is one of the main health challenges faced by most countries around the world, including China. By the end of 2019, China had a population of 253.88 million aged 60 and above, accounting for 18.1% of the total population [1]. As a large agricultural country, China's rural population accounts for a large proportion of the total population. In 2018, the ratio of the elderly aged 65 and over in rural areas (13.84%) was 1.22 times that of urban areas (11.35% ) [2]. In the context of aging, the QoL is an important consideration for the well-being of the elderly.

QoL is a concept closely related to physiology, psychology, social adaptation, and happiness [3]. Compared with survival time, it pays more attention to the life of individuals. A Chinese study showed that restricted by economic development, cultural and educational levels, and medical and health services, the QoL of the elderly in rural areas is poor [4]. Previous studies have linked poor QoL with higher age, unemployment, lower education, insufficient income, illness and poverty [5–7]. Although a lot of research on QoL has been carried out in the elderly, there are few researches on the relationship between functional disability and QoL. For example, several studies on the elderly have found that disability is closely related to the cognitive impairment of the elderly, subjective cognitive impairment is an important factor influencing QoL [8, 9]. At the same time, a review by Djernes showed that the lack or loss of intimacy in social contact is likely to predict depression and low QoL in the elderly [10]. Accordingly, enhancing social roles, participating in collective activities and living with others had a positive effect on promoting the mental and physical health of elderly subjects and improving their QoL [11–13]. QoL is usually an important indicator for the government to implement policies and emphasizes the health and psychosocial welfare issues in the population [14]. Therefore, researching on QoL of the elderly will provide a basis for formulating and implementing appropriate policies and plans to improve QoL of the elderly.

Functional disability of the elderly has become one of the main public health problems faced by many countries [15]. Data from the World Health Survey showed that in countries such as the United States, the United Kingdom, the Netherlands, Sweden, and Switzerland, the increase in functional limitations was mainly manifested in people aged 70 and over [16, 17]. About 15% of the population suffers from functional disabilities to varying degrees around the world [18]. For example, in the Netherlands, the prevalence of functional disability in the elderly is about 22% [19]. And, in the United States, the prevalence of mobility disability among the elderly is 26.9% [20]. In China, according to the data of the second national sample survey of persons with disabilities, the incidence of disability among the population aged 60 and over is about 24% [21]. Based on this calculation, China had 59.95 million elderly people with disabilities in 2019. Many studies at home and abroad have found that the occurrence of disability was related to advanced age [22], weakness [23], depression [24], underweight or overweight, lack of exercise, and low individual health [25]. In addition, many studies had shown that comorbidities are closely related to functional disability [26]. For example, Su found that the elderly in Chinese community, multiple diseases (with chronic diseases) were significantly related to ADL and IADL damage [27].

Studies had shown that functional disability not only impairs the ability of the elderly to live independently, but also lower their QoL [27]. Having trouble with social participation, mobility, social interaction and other disability conditions may have a bad negative influence on the QoL [8, 9]. Health-related variables and basic demographic characteristics such as comorbidities and unemployment are also closely related to lower QoL. However, whether functional disability interacts with these factors and affect the QoL need to be further studied. Improving the QoL for the elderly has become an inevitable requirement of human civilization and social progress. A comprehensive understanding of the factors which affects the QoL is an important reference for planning and implementing medical care and other support programs for the older adults.

In view of this, this study explores the relationship between the six dimensions of functional disability and QoL, and further examines the combined effects of basic demographic characteristics, health-related information and functional disability on the QoL of the rural elderly in Anhui Province.

## Methods

### Study design and data collection

We carried out a cross-sectional study from January to July 2018 in Anhui, China. In order to have a representative sample, we took the multi-stage stratified cluster random sampling method into consideration on the basis of geographical location and economic development. First of all, the three prefecture-level cities of Fuyang (in the north), Hefei (the central, the capital of Anhui) and Anqing (the south) were selected from 16 prefecture-level cities in Anhui. Next, a county was selected randomly in each city. Third, two townships were selected in each county. Last, 3 villages in each selected township were selected randomly, a total of 18 villages were selected as the survey site.

The determination of the sample size in this study was based on the following formula: $N=\frac{Z_{1-\alpha\!\left/2\right.}^2\times P\left(1-P\right)}{E^2}$, α=0.05, ${Z}_{1-\alpha\!\left/2\right.}=1.96$, E=0.2P, P is the proportion of the population with relevant attributes, and 8.8% was taken here (the disability rate of the elderly in rural China), 1-P=0.912. Through this formula, it can be determined that each county needs to sample 995 people, so a total of 2985 people need to be sampled in three counties. Considering the 10% loss to follow-up rate, how many 3284 subjects need to be investigated to meet sample representativeness.

Based on the household registration system data provided by each survey site, 50 households were randomly selected from the list of poor households (the standard for poor households is: the national rural poverty alleviation standard of 2736 yuan per capita net income of farmers in 2013 is used as the identification standard [28]), and randomly selected from poverty 75 non-poor households were selected from neighbors of households for investigation. The subjects of the survey are the resident elderly population (age≥60; living in the place of residence for at least 1 year). With the assistance of local village committees and village doctors, a uniformly trained graduate student of Anhui Medical University conducted a face-to-face investigation on each participant. Before conducting the survey, explain the purpose and procedures of the research to all interviewees, and ensuring that all interviewees have informed consent to this research. For the illiterate interviewees, the informed consent of the guardian was also obtained. Those who were unable to communicate due to cognitive impairment, deafness, etc. are excluded. A total of 3491 older adults participated in the survey, of which 3336 completed the entire survey process, with an effective response rate of 95.56% (3336/3491). This study was ethically approved by the Biomedical Ethics Committee of Anhui Medical University.

### Measurement of Functional Disability

In this study, WHO Disability Assessment Schedule (WHODAS 2.0) was used to assess the functional disability of the survey object. This scale contained 36 items that measure six dimensions of cognition, mobility, self-care, getting along, life activities and social participation. Each item had five options, with scores ranging from 1 to 5 and respondents were asked to rate their agreement (1 = “No difficulty”, 2 = “Slightly difficult”, 3 = “Moderately difficult”, 4 = “Severely difficult”, 5 = “Extremely difficult”). According to the manual of the scale [29], the original scores were converted, and each dimension and total scores were calculated. The total score was 0-100 (0 points = “no disability”, 100 points = “complete disability”). According to the ICF (International Classification of Functioning, Disability and Health) standard for evaluation, <4 is classified as robust, and >4 is classified as mild or above disability (limited) [30].

### Measurement of Quality of Life

The QoL was assessed using the Chinese version of the EQ-5D-3L scale (Five-Dimensional European Quality Of Health Scale), which was confirmed had good reliability and validity in a Chinese elderly sample [30]. The scale is composed of EQ-5D health state description system and EQ-VAS. The five dimensions of health state description system encompassed mobility, self-care ability, usual activities, pain/discomfort, and anxiety/depression. There are three options in each dimension, with scores ranging from 1 to 3 and respondents were asked to rate their agreement (1= “no problems”, 2= “moderate problems”, 3= “extreme problems”). EQ-VAS is a visual scale of 20 cm, ranging from 0 (representing the worst health in the mind) to 100 (representing the best health in the mind), respondents use the most appropriate point on the visual scale to evaluate their day health status.

Considering the ceiling effects of EQ-5D-3L [31], we regrouped the three options (no problems, moderate problems and extreme problems) into two groups, called no problems and any problems. The total score of QoL was calculated according to the Japanese scale utility scoring system [32]. The total utility score between -0.111 to 1 [33]. If the total score of QoL for a participant is 1, he/she must all choose “no problems” among the above 5 dimensions, so QoL can be divided into two categories: good and poor. Namely, respondents who choose at least one “any problems” were defined as having a poor QoL, others were good.

### Demographic Characteristics

Others variables consist of basic demographics and health-related information. Specifically include the followings: age (60-69, 70-79, ≥80 years), gender (female, male), education level (illiterate, Primary and above), employment status (unemployed, employed), living style (living alone, living with spouse, other), region (northern, central, southern of Anhui Province), source of income (employment income, child support, government subsidy). Based on the China's poverty standard: families whose annual per capita income is below the national poverty line and were recognized by the Office of Poverty Alleviation, poverty (yes, no) of the participants was obtained. Information on physical discomfort (within two weeks), hospitalization (within one year), number of chronic diseases was also collected.

### Statistical analysis

First of all, we used Chi-squared test to test the difference between different QoL groups (good and poor). Rate and percentage were employed to describe the demographic characteristics of the participants between different groups.

Next, the relationship between QoL and functional disability were investigated in terms of binary logistic regression model. The results were expressed with the odds ratio (OR) and associated 95% confidence interval (95% CI). Then, according to the literature review, related variables such as age, gender, living status, education level, region, employment status, number of chronic diseases, and so on were adjusted in regression models. And we got adjusted odds ratio (AOR) and associated 95% confidence interval (95% CI). According to the results of the variance expansion coefficient (VIF), there is no evidence of multicollinearity in the model, and no factors exceeding the critical value (Table S1).

Lastly, in order to further study the interaction between disability and related factors related to QoL, classification and regression tree models (CART) were used. It can check complex combinations or interactions between factors and variables which may be overlooked in traditional analysis methods [34]. This model also can be used to explore some homogenous subgroups related to the development of QoL. The variables contained in this model were on the base of the unadjusted results of the previous binary logistic regression. For the purpose of having the optimal model, the growth method we chose was exhaustive CHAID. And maximum growth depth was set 3.

All statistical analysis used SPSS statistical software, version 23. Two-tailed p < 0.05 was considered statistically significant.

## Results

### Characteristics of participants

Table 1 describes the general demographic characteristics of the respondents. The study involved a total of 3336 subjects, of which 621 subjects had a high QoL, and the remaining 2715 subjects had a low QoL. There were significant statistical differences between the two groups of survey subjects in basic demographic characteristics and functional disability dimensions (*P*-value<0.05). Among the 621 subjects with high QoL, 59.6% (370/621 persons) were men, 57.5% (358/621 persons) were between 60-69 years old, and 44.4% (276/621 persons) were living with partners, 84.2% (523/621 people) have not been hospitalized in the past year, and 84.4% (524/621 people) income is from their own work. At the same time, 43.3% (269/621 people) of the survey respondents with high QoL did not have any chronic diseases.

**Table 1:** General characteristics of the respondents (N = 3336)

	Total	Quality of Life		χ^2^	*p*-value
	(N=3336)	Good(N=621)	Poor(N=2715)		
**Gender**				33.153	<0.001
Male	1640(49.2)	370(59.6)	1270(46.8)		
Female	1696(50.8)	251(40.4)	1445(53.2)		
**Age(years)**				65.987	<0.001
60–69	1529(45.8)	358(57.6)	1171(43.1)		
70–79	1325(39.7)	228(36.8)	1097(40.4)		
≥80	482(14.5)	35(5.6)	447(16.5)		
**Education level**				25.414	<0.001
Illiterate	2211(66.3)	358(57.7)	1853(68.3)		
Primary and above	1125(33.7)	263(42.3)	862(31.7)		
**Employment status**				111.305	<0.001
Unemployed	2137(64.1)	284(45.7)	1853(68.3)		
Employed	1199(35.9)	337(54.3)	862(31.7)		
**Living style**				7.920	0.019
Living alone	660(19.8)	98(15.8)	562(20.7)		
Living with spouse	1436(43.0)	276(44.4)	1160(42.7)		
Other	1240(37.2)	247(39.8)	993(36.6)		
**Region**				57.991	<0.001
Northern	1482(44.4)	198(31.9)	1284(47.3)		
Central	919(27.5)	184(29.6)	735(27.1)		
Southern	935(28.1)	239(38.5)	696(25.6)		
**Poverty**				42.604	<0.001
Yes	1206(36.2)	154(24.8)	1052(38.7)		
No	2130(63.8)	467(75.2)	1663(61.3)		
**Physical discomfort**				178.701	<0.001
**(Within two weeks)**					
Yes	2120(63.5)	250(40.3)	1870(68.9)		
No	1216(36.5)	371(59.7)	845(31.1)		
**Chronic diseases**				164.175	<0.001
0	865(25.9)	269(43.3)	596(22.0)		
1	1227(36.8)	239(38.5)	988(36.3)		
≥2	1244(37.3)	113(18.2)	1131(41.7)		
**Hospitalization**				90.620	<0.001
**(Within a year)**					
Yes	1062(31.8)	98(15.8)	964(35.5)		
No	2274(68.2)	523(84.2)	1751(64.5)		
**Source of income**				23.457	<0.001
Employment income	2569(77.0)	524(84.4)	2045(75.3)		
Child support	395(11.8)	51(8.2)	344(12.7)		
Government subsidy	372(11.2)	46(7.4)	326(12.0)		
**Cognition**				32.797	<0.001
Limited	2312(69.3)	371(59.7)	1941(71.5)		
Robust	1024(30.7)	250(40.3)	774(28.5)		
**Mobility**				441.426	<0.001
Limited	2197(65.9)	185(29.8)	2012(74.1)		
Robust	1139(34.1)	436(70.2)	703(35.9)		
**Self-care**				183.178	<0.001
Limited	988(29.6)	45(7.2)	943(34.7)		
Robust	2348(70.4)	576(92.8)	1772(65.3)		
**Getting along**				165.945	<0.001
Limited	1253(37.6)	93(15.0)	1160(42.7)		
Robust	2083(62.4)	528(85.0)	1555(57.3)		
**Life activities**				440.374	<0.001
Limited	2170(65.0)	179(28.8)	1991(73.3)		
Robust	1166(35.0)	442(71.2)	724(26.7)		
**Social participation**				286.526	<0.001
Limited	3012(90.3)	448(72.1)	2564(94.4)		
Robust	324(9.7)	173(27.9)	151(5.6)		

### Results of logistic regression analysis

Table 2 shows the relationship between QoL and functional disability. In a univariate model, compared with the control group, as age increases, the likelihood of a lower QoL is greater. In women (OR=1.677, 95%CI: 1.405-2.002), age ≥80 years (OR=3.905, 95%CI: 2.714-5.618), with ≥2 chronic diseases (OR=4.517, 95%CI: 3.550-5.749), and the population whose main economic income is government subsidy (OR=1.816, 95%CI: 1.314-2.509) have the highest QoL. People with higher education level, living with others, from southern Anhui, not in poverty, not discomfort in the past 2 weeks, and those who have not been hospitalized in the past one year may have a higher QoL. Compared with the control group, in the six functional disability dimensions, all disability states are associated with lower QoL.

**Table 2:** Logistic regression analysis examining the association between disability and QOL (N =3336)

	Crude				Adjusted			
	B	S.E.	Wals	OR(95%CI)	B	S.E.	Wals	OR(95%CI)
**Gender**								
Male				(reference)				(reference)
Female	0.517	0.090	32.749	1.677(1.405,2.002)***	0.080	0.116	0.476	1.083(0.863,1.360)
**Age**								
60-69				(reference)				(reference)
70-79	0.386	0.095	16.649	1.471(1.222,1.771)***	-0.092	0.114	0.650	0.912(0.730,1.140)
≥80	1.362	0.186	53.849	3.905(2.714,5.618)***	0.288	0.222	1.684	1.333(0.863,2.059)
**Education level**								
Illiterate				(reference)				(reference)
Primary and above	-0.457	0.091	25.169	0.633(0.530,0.757)***	-0.110	0.117	0.897	0.895(0.712,1.125)
**Living style**								
Living alone				(reference)				(reference)
Living with spouse	-0.311	0.128	5.864	0.733(0.570,0.942)*	-0.003	0.157	0.000	0.997(0.733,1.355)
Other	-0.355	0.131	7.404	0.701(0.543,0.905)**	-0.026	0.165	0.025	0.974(0.705,1.345)
**Region**								
Northern				(reference)				(reference)
Central	-0.485	0.112	18.596	0.616(0.494,0.768)***	-0.376	0.143	6.871	0.687(0.519,0.910)**
Southern	-0.801	0.107	55.976	0.449(0.364,0.554)***	-0.496	0.134	13.760	0.609(0.469,0.791)***
**Poverty**								
Yes				(reference)				(reference)
No	-0.651	-0.101	41.660	0.521(0.428,0.635)***	-0.276	0.127	4.714	0.758(0.591,0.973)*
**Physical discomfort(Within two weeks)**								
Yes				(reference)				(reference)
No	-1.189	0.092	168.056	0.304(0.254,0.364)***	-0.557	0.110	25.617	0.573(0.462,0.711)***
**Hospitalization(Within a year)**								
Yes				(reference)				(reference)
No	-1.078	0.117	84.635	0.340(0.271,0.428)***	-0.367	0.134	7.496	0.693(0.533,0.901)**
**Number of chronic diseases**								
0				(reference)				(reference)
1	0.642	0.103	36.726	1.866(1.525,2.283)***	0.179	0.121	2.177	1.196(0.943,1.517)
≥2	1.508	0.123	150.299	4.517(3.550,5.749)***	0.708	0.144	24.073	2.030(1.530,2.693)***
**Source of income**								
Employment income				(reference)				(reference)
Child support	0.547	0.158	12.017	1.728(1.268,2.335)**	-0.055	0.194	0.079	0.947(0.648,1.384)
Government subsidy	0.597	0.`165	13.084	1.816(1.314,2.509)***	-0.200	0.215	0.868	0.818(0.537,1.248)
**Employment status**								
Unemployed				(reference)				(reference)
Employed	-0.936	0.090	107.089	0.392(0.328,0.468)***	-0.644	0.118	29.777	0.525(0.417,0.662)***
**Cognition**								
Robust				(reference)				(reference)
Limited	1.130	0.073	241.337	1.690(1.410,2.025)***	-0.739	0.127	33.841	0.477(0.372,0,613)***
**Mobility**								
Robust				(reference)				(reference)
Limited	1.909	0.098	378.810	6.745(5.566,8.175)***	**0.808**	**0.129**	**39.484**	2.243(1.743,2.885)***
**Self care**								
Robust				(reference)				(reference)
Limited	1.919	0.160	143.892	6.812(4.979,9.320)***	0.301	0.217	1.926	1.352(0.883,2.068)
**Getting along**								
Robust				(reference)				(reference)
Limited	1.443	0.119	147.227	4.235(3.354,5.347)***	**0.480**	**0.164**	**8.604**	1.615(1.173,2.226)**
**Life activities**								
Robust				(reference)				(reference)
Limited	1.916	0.099	377.008	6.791(5.597,8.239)***	**0.914**	**0.131**	**48.397**	2.494(1.928,3.226)***
**Social participation**								
Robust				(reference)				(reference)
Limited	1.881	0.123	235.370	6.557(5.157,8.338)***	**0.797**	**0.149**	**28.523**	2.218(1.656,2.971)***

*p < 0.05; **p < 0.01; ***p < 0.001

After adjusting all covariates (gender, age, education level, living style, region, poverty, physical discomfort within two weeks, hospitalization within one year, number of chronic diseases, source of income, employment status), compared with the reference group, had limited in dimension of mobility (AOR=2.243, 95%CI:=1.734-2.885), getting along (AOR=1.615, 95%CI: 1.173-2.226), life activities (AOR=2.494, 95% CI: 1.928-3.226), social participation (AOR=2.218, 95% CI: 1.656-2.971) are related to QoL, which shows that those who had limited in mobility, getting along, life activities, and social participation are risk factors for QoL. However, the cognition dimension is a protective factor for QoL. In addition, the adjusted model did not show any significant statistical correlation between self-care and QoL.

### Results of classification and regression tree model

**Fig. 1:**
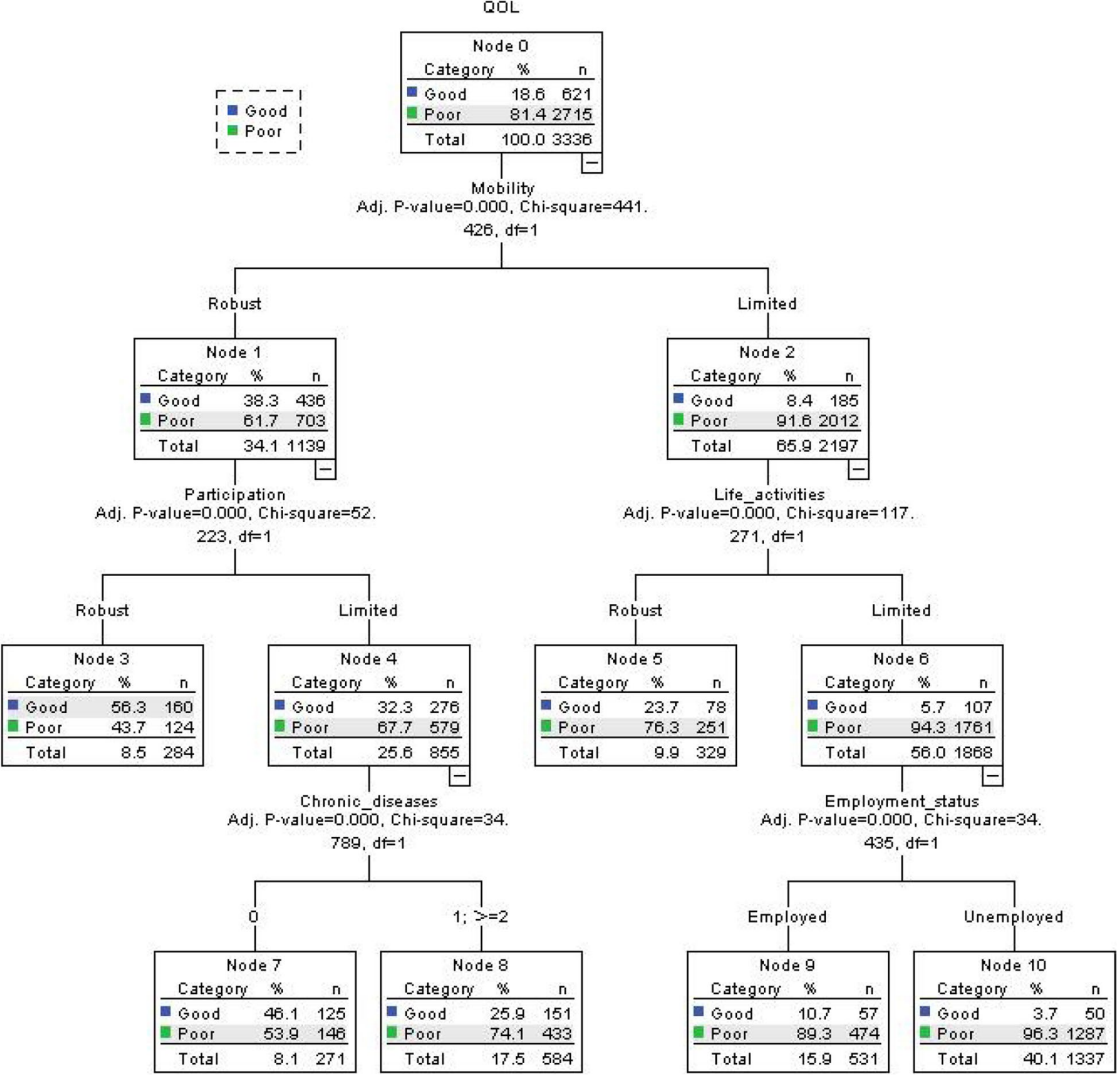
Classification and regression tree model (N=3336)

The results of CART model are displayed in Figure 1. QoL is mainly related to mobility, social participation, life activities, number of chronic disease, and employment status. Mobility is the primary factor related to the QoL. Furthermore, the interaction between the dimensions of functional disability and the variables were identified.

Those elderly who had mobility limited (node2), life activities limited (node6), unemployed (node10) characteristics may have a lower QoL.

Among participants with no chronic disease (node7), limited in social participation (node4), in comparison with chronic disease (node8), the possibility of suffering from low QoL was higher.

For the elderly with limited mobility (node 1) and limited social participation (node 3), the incidence of high QoL is higher.

## Discussion

This study examined the relationship between the disability status and the QoL of the elderly in rural Anhui. The results revealed a correlation between disability status and QoL, as well as the combined effect of mobility, social participation, life activities dimensions of functional disability and number of chronic diseases and employment status on QoL. In particular, after adjusting the relevant covariates, the QoL of the elderly with mobility, getting along, life activities, and social participation limited may be lower.

### Social Participation and QoL

The result of the binary logistic regression analysis of this study showed that whether before or after the covariate adjustment, the rural elderly social participation was significantly related to the QoL. Specifically, the higher level the elder whose social participation was, the better the QoL. Which is in line with a Australia’s structured interview with Indigenous people over 45 years old living in Australia and Melbourne [35], the result showed that establishing contact with the community and regularly participating in community social activities are essential to improve the QoL. Tak et al. pointed out in meta-analysis that appropriate participation of the elderly population can delay the aging process, reduce the incidence of disability, and improve the QoL [36]. The possible explanation for this result is that as China's urbanization continues to deepen [37], more and more young laborers are moving to cities, and the rate of rural elderly living alone is increasing. These elderly people who lack emotional and physical support, prone to depression, manifested as loss of interest in life, inattention, loss of appetite, and even increased risk of suicide [38], resulting in a decline in the QoL. Increasing social participation can reduce loneliness, produce positive emotions, and avoid depression. To this end, communities should increase health promotion-related activities and actively encourage elderly people to strengthen social participation.

### Mobility and QoL

Mobility is another important factor that affected the QoL. In other words, for the elderly compared with those mobility limited, the QoL of the robust is better. Many factors, such as loneliness and reduced mobility, lead to poor mental health and cognitive function in the elderly, thereby affecting the QoL [39]. A Turkish study of elderly people aged 65 and above found consistent with our results [40], suggesting that engaging in leisure activities has an important significance in improving the QoL. Chronic disease is an important factor affecting the QoL of the elderly. Leisure activities and physical exercises have a preventive and control effect on chronic diseases, thereby improving the QoL [41]. Relatively complete mobility can promote the social participation of the elderly, such as communicating with neighbors and participating in activities. In this process, the elderly can get along with others, talk about their concerns, gain communication, avoid the adverse effects of loneliness on the cardiovascular system, immune system and physical and mental health [42], then improve the QoL [35].

### Life activites and QoL

Older subjects with life activities limited had a lower QoL. Life activities includes a variety of housework activities and work assignments, which can increase the daily exercise opportunities for the elderly. A 12-month intervention study conducted by Naoto Taguchi and others in Japan on the elderly (median age 84 years) found that exercise can improve people's physical functions and health-related QoL [43]. Studies have shown that the level of physical activity is related to the QoL, and the QoL can be improved after exercise [44]. The QoL of elderly subjects with life activities robust may be higher, the possible reason is that the elderly can gain recognition from family members and others when performing daily activities, and increase their sense of self-efficacy [45]. Elderly people with high self-efficacy are more confident in dealing with problems. They are more likely to solve difficulties and problems by improving their own abilities and formulating more effective measures and maintain a positive mental state [46]. A study found that those who positive outlook on their lives, having a moderately positive QoL [47].

### Getting along and QoL

Among the rural elderly population, those with limited getting along dimensions have a worse QoL than those with robust. This result is similar to a cross-sectional study in Poland that shows that getting along with others is associated with a higher QoL [48]. At the same time, it is reported that the QoL of the elderly with 6 or more close friends is higher than that of the elderly population without friends [49]. Some studies also show that keeping in touch with others can reduce the impact of psychological problems, which helps improve the QoL [50]. The possible explanation for this result is that as the age increases, the elderly need to keep in close contact with family, friends, and neighbors in order to obtain information and keep in touch with the ever-changing external world. These meaningful external ties challenge the elderly to understand their surroundings and values in their society [49], which may result in better social participation and conducive to improving health and QoL [51].

### Cognition and QoL

Interestingly, an interesting phenomenon was found in this study. For the QoL, cognition is a protective factor. Specifically, the QoL of the elderly with cognition limited may be higher, which is different from previous studies. A Korean study showed that in elderly women, the QoL is more related to cognitive function [39]. Studies such as Leonardo have shown that subjective cognitive impairment is common in the elderly and affects the QoL [7]. 83% of cognitively impaired elderly people experience behavioral symptoms, and these behaviors and emotional states often have a negative impact on the QoL [52]. The reason may be that the area covered by the village as a unit is relatively small. Most of the villagers lived in a concentrated manner, they have closely contacts in daily life [33]. The neighbors understood each other and helped each other. The elderly with cognition limited were given more care. In addition, the Chinese government has a corresponding security system and economic subsidies for the elderly who have no financial resources and have difficulties in life, so that the basic life of the elderly with cognition limited can be guaranteed.

### Self-care and QoL

For the self-care dimension, before adjusting the related variables, the QoL of limited persons was lower than that of the elderly with self-care robust, but after adjustment, this correlation did not have significant statistical significance. This is inconsistent with the research results of Donnapa Hongthong et al. They found that Activity Daily Living (ADL) of the elderly has an important influence on QoL [53]. Somrongthong's study on the QoL of 400 elderly people in rural areas in northeastern Thailand also found that there is a statistically significant correlation between ADL restriction and QoL [54]. The possible explanation for the inconsistency between this research and the above research results is: elderly people who have difficulties in bathing, dressing, eating, etc. generally live with their children or other people. And live with their families, not only can they receive daily care, but more importantly, companionship and psychological support. Relevant studies have shown that among the rural population, the QoL of the elderly living with their children or companions was better than that of those living alone [55]. The elderly with self-care robustness may lack companionship, but with ADL in a complete state, the QoL will be better [56]. Therefore, there is no statistical difference in the QoL of the elderly with self-care.

### Interaction relationships on QoL

In this study, the interaction between functional disability and other related variables and QoL was explored. Previous studies have found that unemployment, illness, and low education were associated with a high risk of low QoL for the elderly [5, 6]. At the same time, the Agnieszka study pointed out that the elderly population suffered from a high incidence of chronic diseases and disability, which affected their QoL and revealed the relationship between disability and QoL [48]. In our research, more importantly, we used the CART model to observe the interaction between functional disability, the number of chronic diseases, and whether or not to work. Specifically, the QoL of the elderly population in mobility limited, life activities limited, and unemployed may be lower. This shows that mobility and life activities as dimensions of disability may be closely related to improve the QoL. In addition, those with less social participation were more likely to have a lower QoL than elderly subjects with higher social participation. The above can show the advantages of the CART model, and you can find interaction relationships that were not observed in the binary logistic regression analysis. We demonstrated the interaction between functional disability, other related variables and QoL, and found out which factors and characteristics superimposed elderly subjects are high-risk groups with low QoL. These findings may have positive significance for formulating and standardizing corresponding health policies, improving the QoL of the elderly, and promoting the development of healthy aging.

### Strengths and limitations

Our research has the following advantages. First of all, the effective response rate of this study is 95.56% (3336/3491). As we all know, the results of studies with a high effective response rate are more reliable. Second, we introduced the CART model in the data analysis. This model can comprehensively explore the interaction between multiple variables, effectively avoid the possible collinearity between variables, automatically classify according to the significance level of the test, and pass the intuitive tree state chart display. Finally, this study explored the relationship between functional disability and other variables related to the QoL of rural elderly people. This provides a basis for formulating targeted improvement and preventive measures to improve the QoL of the elderly.

However, this study also has the following limitations. First of all, because this study is a cross-sectional study, although it is found that there is a correlation between functional disability and QoL, it is difficult to judge the causal relationship. This is what we need to pay attention to in the future research design. Secondly, the survey objects of this study only cover rural areas in Anhui Province. Affected by factors such as economic development and cultural background, the extension of the results of this study is limited.

## Conclusion

This study provides a basis for the relationship between QoL and functional disability, indicating that functional disability is related to QoL. Specifically, the elderly with limited mobility, getting along, life activities, social participation and those with cognitive robustness are more likely to have low QoL. Our research may provide reference for formulating plans and measures to improve the QoL of the elderly, and promote the active and healthy development of aging.

## Supplementary Information

**Additional file 1.**

## Data Availability

Not applicable. The datasets generated during the study are not publicly available due to an ethical restriction but are available from the corresponding author on reasonable request.

## Abbreviations

QoL: Quality of life
WHODAS 2.0: WHO Disability Assessment Schedule
EQ-5D-3L: Five-Dimensional European Quality Of Health Scale
CART: Classification and regression tree model
