# Supplementary material for: Correlation between functional disability and quality of life among rural elderly in Anhui province, China: a cross-sectional study
Source: BMC Public Health. 2022 Feb 25;22:397. doi: 10.1186/s12889-021-12363-7 (PMC8881859; doi:10.1186/s12889-021-12363-7)
Supplement: Supplementary file 1 — Additional file 1. [file 12889_2021_12363_MOESM1_ESM.pdf]

TableS1 Results of multicollinearity examination of variables

variables	Non-standardized coefficient		Standard coefficient	t-Value	Sig.	Collinearity statistics	
	B	Standard error				Tolerance	VIF
(constant)	1.422	.084		16.885	.000		
Source of income	-.010	.010	-.018	-1.026	.305	**.762**	**1.313**
Cognition	-.092	.015	-.109	-6.249	.000	**.751**	**1.332**
Mobility	.127	.017	.155	7.701	.000	**.568**	**1.760**
Self care	-.006	.019	-.007	-.333	.739	**.461**	**2.169**
Getting along	.047	.017	.058	2.708	.007	**.498**	**2.008**
Life activities	.140	.017	.171	8.427	.000	**.557**	**1.794**
Participation	.179	.022	.137	8.014	.000	**.793**	**1.261**
Chronic diseases	.039	.008	.078	4.724	.000	**.842**	**1.188**
Hospitalization within a year	-.028	.013	-.034	-2.121	.034	**.897**	**1.115**
Physical discomfort within 2 weeks	-.075	.013	-.093	-5.605	.000	**.841**	**1.190**
Poverty	-.025	.013	-.031	-1.902	.057	**.847**	**1.180**
Region	-.029	.008	-.061	-3.751	.000	**.861**	**1.161**
Living style	.003	.009	.006	.352	.725	**.864**	**1.157**
Employment status	-.080	.014	-.099	-5.740	.000	**.771**	**1.297**
Education level	-.022	.014	-.027	-1.608	.108	**.815**	**1.227**
Age	.005	.009	.009	.555	.579	**.794**	**1.259**
Gender	.006	.013	.007	.429	.668	**.792**	**1.263**

a. Dependent variable\: QOL
